# Conservation of *ParaHox *genes' function in patterning of the digestive tract of the marine gastropod *Gibbula varia*

**DOI:** 10.1186/1471-213X-10-74

**Published:** 2010-07-12

**Authors:** Leyli Samadi, Gerhard Steiner

**Affiliations:** 1 Molecular Phylogenetics, Department of Evolutionary Biology, Faculty of Life Sciences, University of Vienna, Vienna, Austria

## Abstract

**Background:**

Presence of all three *ParaHox *genes has been described in deuterostomes and lophotrochozoans, but to date one of these three genes, *Xlox *has not been reported from any ecdysozoan taxa and both *Xlox *and *Gsx *are absent in nematodes. There is evidence that the *ParaHox *genes were ancestrally a single chromosomal cluster. Colinear expression of the *ParaHox *genes in anterior, middle, and posterior tissues of several species studied so far suggest that these genes may be responsible for axial patterning of the digestive tract. So far, there are no data on expression of these genes in molluscs.

**Results:**

We isolated the complete coding sequences of the three *Gibbula varia ParaHox *genes, and then tested their expression in larval and postlarval development. In *Gibbula varia*, the *ParaHox *genes participate in patterning of the digestive tract and are expressed in some cells of the neuroectoderm. The expression of these genes coincides with the gradual formation of the gut in the larva. *Gva-Gsx *patterns potential neural precursors of cerebral ganglia as well as of the apical sensory organ. During larval development this gene is involved in the formation of the mouth and during postlarval development it is expressed in the precursor cells involved in secretion of the radula, the odontoblasts. *Gva-Xolx *and *Gva-Cdx *are involved in gut patterning in the middle and posterior parts of digestive tract, respectively. Both genes are expressed in some ventral neuroectodermal cells; however the expression of *Gva-Cdx *fades in later larval stages while the expression of *Gva-Xolx *in these cells persists.

**Conclusions:**

In *Gibbula varia *the *ParaHox *genes are expressed during anterior-posterior patterning of the digestive system. This colinearity is not easy to spot during early larval stages because the differentiated endothelial cells within the yolk permanently migrate to their destinations in the gut. After torsion, *Gsx *patterns the mouth and foregut, *Xlox *the midgut gland or digestive gland, and *Cdx *the hindgut. *ParaHox *genes of *Gibbula *are also expressed during specification of cerebral and ventral neuroectodermal cells. Our results provide additional support for the ancestral complexity of *Gsx *expression and its ancestral role in mouth patterning in protostomes, which was secondarily lost or simplified in some species.

## Background

The three *ParaHox *genes, *Gsx*, *Xlox*, and *Cdx*, were first described as a gene cluster in the invertebrate chordate *Branchiostoma floridae *(amphioxus) by the elegant work of Brooke et al. 1998 [1]. *ParaHox *and *Hox *genes are believed to have evolved from a single ancient proto-*Hox *cluster composed of two to four genes prior to the divergence of cnidarians and bilaterians. Thus, they are considered evolutionary sister (or paralogue) clusters [1-7]. Vectorial expression of the *ParaHox *genes in anterior, middle, and posterior tissues of amphioxus and its distinct similarities to vertebrate *ParaHox *gene expression suggest that these genes may be responsible for axial patterning of the digestive tract [1,3].

### Expression of *ParaHox *genes in deuterostomes

*ParaHox *gene expression and genomic organisation have been studied extensively in deuterostomes. In Vertebrates, *Gsh1 *and *Gsh2 *genes are restricted to the central nervous system (CNS) [8-13]. Vertebrate *Xlox *is expressed both in CNS and the developing gut [14-20]. *Cdx1 *to *Cdx4 *genes of vertebrates are involved in posterior patterning, since they are expressed in posterior parts of CNS and gut [21-24]. Within invertebrate deuterostomes, apart from amphioxus, expression of *ParaHox *genes has been traced in the ascidian, *Ciona intestinalis*, the echinoderm, *Strongylocentrotus purpuratus*, and in a starfish, *Archaster typicus *[25-29]. In invertebrate deuterostomes, *Gsx *is expressed more anteriorly and only in the nervous system, while *Xlox *and *Cdx *are expressed within the gut primordium with *Xlox *anterior to *Cdx *[25-28]. In the sea star, however, the *Aty-Xlox *expression is found in the archenteron as well as in ectodermal cells near the vegetal region of early and mid-gastrula stages [29]. This expression pattern is very different from those of *Xlox *homologues in other deuterostomes.

### Expression of *ParaHox *genes in ecdysozoans

In ecdysozoans, *Gsx *expression has been documented in the insects *Drosophila *and *Tribolium *[30,31]. Insect *Gsx *(called *ind*) is expressed along a pair of medio-lateral neural columns and promotes neural precursor formation in the medial and intermediate columns of the CNS [30,31]. The central *ParaHox *gene, *Xlox*, is lost in all insect genomes sequenced to date. *Caudal *has been known as a posterior patterning gene in several arthropods during segmentation [32-42]. *Cdx *is also a posterior patterning gene in the nematode *Caenorhabditis elegans*. Here, this gene is called *pal-1 *and patterns the precursor cells of alae and rays in the posterior of the worm [43]. *Gsx *and *Xlox *orthologs are absent in the nematode [44].

### Expression of *ParaHox *genes in lophotrochozoans

Within Lophotrochozoa, expression of the full complement of *ParaHox *genes has been described in the polychaetes *Capitella teleta*, *Nereis virens*, and *Platynereis dumerilii *[45-48]. In *Capitella, Gsx *is not expressed in the gut but in some neuroectoderm cells of the anterior brain [45]. This is very different from the expression of *Gsx *in the nereid polychaetes, *Nereis virens *and *Platynereis dumerilii *[46,48]. Nereid *Gsx *is first expressed in symmetrical bilateral domains in the dorso-medial episphere of the trochophore [46,48]. Later this gene is expressed during formation of the midgut and the posterior foregut in both nereids [46,48]. *Xlox *is expressed throughout the midgut in the polychaete *Capitella *[45]. This is also true for the *Xlox *genes, named *Lox3 *in the leeches *Helobdella triserialis *and *Hirudo medicinalis *[49,50]. Expression of *Xlox *is not reported in the nervous system of these annelids [45,49,50]. Nereid *Xlox *is also expressed in the midgut, but in contrast to *Capitella *and the leeches its expression is additionally detected in the CNS [46,48]. As in arthropods, *Cdx *is a posterior patterning gene in the annelids. However in *Platynereis*, *Nereis*, *Tubifex*, and *Capitella *there are both anterior and posterior expression domains of *Cdx *[45-48,51]. *Capitella Cdx *is expressed in the cerebral ganglia, *Nvi-Cdx *expression is detectable in the ventral nervous system, while *Pdu-Cdx *is not detected in the nervous system [45-48]. Expression of *Cdx *is also detected in more posterior parts of the gut [45-48]. Moreover, *Cdx *is expressed in the posterior ectodermal cells that form the pygidium epidermis of both nereids [46-48]. Additionally, *Cdx *expression can be traced in mesodermal cells in *Capitella*, *Tubifex*, and *Platynereis *[45,47,48,51].

Little is known of *ParaHox *genes in other Lophotrochozoa than annelids [52-55]. The only available data are on the *Cdx *gene expression during the early development of the marine limpet, *Patella vulgata *[53]. *Pvu-Cdx *is expressed at the onset of gastrulation in the ectodermal cells at the posterior edge of the blastopore and in the paired mesentoblasts [53]. During trochophore larval stage, *PvuCdx *is expressed in the posterior neurectoderm of the larva, as well as in part of the mesoderm [53]. Within Mollusca, a full complement of *ParaHox *genes has been shown for the chiton *Nuttallochiton mirandus *and the scallop *Pecten maximus *[54,55]. However the information is limited to partial homeobox sequences, whereas expression patterns of *Gsx *and *Xlox *or chromosomal organisation of *ParaHox *genes have not been reported yet in any mollusc species.

### Ancestral role of *ParaHox *genes

Holland (2001) elaborated the hypothesis of the ancestral role of *ParaHox *genes proposed by the original work of Brooke et al. 1998 [1,3]. Holland's hypothesis proposes that the three *ParaHox *genes originated from the Proto *Hox *gene cluster and pattern anterior, middle and posterior gut regions in a colinear manner in basal animals [3]. According to this hypothesis, a link of *Gsx *and anterior gut development existed in basal animals. However, *Gsx *is not expressed in the anterior gut of deuterostomes. This is explained by the loss of the primary mouth and formation of a secondary mouth in deuterostomes [3].

### Aim

Gastropoda is undoubtedly the most successful taxon of the Mollusca, embracing more than 80% of all mollusc species [56]. The vetigastropod *Gibbula varia *L. is a shallow subtidal top shell snail with encapsulated development. The lecithotrophic larval development is completed within the eggs. The juveniles leave the gelatinous egg masses only after metamorphosis. In order to elucidate the function of the *ParaHox *genes in molluscs and to gain broader insights into the evolution of the *ParaHox *genes in the Lophotrochozoa, we describe the sequences as well as expression patterns for all three *ParaHox *orthologues by whole mount in situ hybridization from embryonic through juvenile stages in the top shell *Gibbula varia*. This is the first report of expression patterns of the full *ParaHox *complement in a mollusc.

## Results

### *Gibbula varia *life history

*Gibbula varia *is a dioecious species. The eggs fertilized via copulation are laid in gelatinous egg masses (additional file 1, Figure S1A, S1B, S1C). Development of the embryos and larvae takes place inside the egg capsule and takes about four days. The main stages of *G. varia *development are presented in Table 1. Epibolic gastrulation occurs by the micromeres rapidly spreading downwards and enclosing the macromeres. The blastopore, being wide at first, gradually becomes constricted at 10 to 12 hours post fertilization (hpf), when the trochoblasts start to become ciliated. At 16 hpf, the prototroch is clearly visible as a circular ciliary band, separating the trochophore larva's episphere from the hyposphere (additional file 1, Figure S1D). There is no sign of apical cilia (apical tuft) at any stage in the development of the trochophore, although the pretrochal cells were observed to be smaller than those of the posttrochal region (additional file 1, Figure S1D). By this stage the blastopore gradually moves to a position just below the prototroch, forming the stomodeum. Simultaneously, the shell-gland invagination appears as a thin patch of cells gradually spreading over the dorsal region of the larva (additional file 1, Figure S1D). At 18 hpf, the late trochophore larva comprises a prototroch, the shell field surrounded by the mantle edge, and a pedal rudiment (additional file 1, Figure S1E). The late trochophore (24 hpf) turns into an encapsulated pretorsional veliger larva by differentiation of the prototroch to a distinct velum (additional file 1, Figure S1F). The mantle fold and mantle cavity become visible mid-ventrally on the posterior surface of the pedal rudiment (additional file 1, Figure S1E, S1G). At 36 hpf, the pretorsional veliger has a velum, an apical organ marked by apical cilia (apical tuft), a mouth opening, and a pedal rudiment with the operculum anlage (additional file 1, Figure S1H). The first 90° of torsion take place between 36-48 hpf, presumably by contraction of the larval retractor (shell) muscle. This results in a 90° displacement of the mantle cavity to the right side, and, when viewed from the front, the foot and velum are rotated anti-clockwise in relation to the protoconch. The remaining part of torsion is completed within one day while the velum gradually becomes reduced in size and splits ventrally (additional file 1, Figure S1I). At 60 hpf, the operculum appears in the posttorsional veliger larva (additional file 1, Figure S1I). The radula and cephalic eyes appear about three days after fertilization. As the eyes form, the cephalic tentacles begin to appear as outgrowths of the prevelar surface. The juvenile hatches on the fourth day of development (about 96 hpf) and after that mineralization of the shell begins. The animals become sexually mature after 11-12 months.

**Table 1:** Timing of developmental stages of *Gibbula varia *(at 22°C); different stages of larval development and metamorphosis of *G. varia *inside the gelatinous egg capsules before hatching.

Name of stage and approximate time of development (hpf)	Brief description of main features
Early Trochophore Larva (12 hpf)	The pretrochal cells are smaller than the posttrochal cells; prototroch starts to form by cilliation of trochoblasts; shell gland starts to evaginate; foot rudiment and stomodaeum are not completely formed.
Late Trochophore Larva (18-24 hpf)	The larva comprises a prototroch, shell field surrounded by mantle edge, a pedal rudiment, and stomodaeum.
Pretorsional veliger larva (36-48 hpf)	The mantle and mantle cavity form. The larva has a velum, apical organ marked by apical cilia, mouth opening, and pedal rudiment with anlage of operculum.
Post-torsional veliger larva (60 hpf)	The mantle lies over the back of the head and the velum gradually splits ventrally, the operculum apears.
Metamorphotic (competence) stage (72 hpf)	Eye rudiments and cephalic tentacles begin to form in the prevelar area. The anlage of the radula becomes visible.
Encapsulated juvenile	Velum is completely lost; eyes and cerebral tentacles are formed.
Hatchling (96 hpf)	The encapsulated juvenile hatches and shell mineralization begins.

### Development of gut in *G. varia*

The development of the digestive tract starts with the development of the stomodeum (future mouth opening) in the trochophore (additional file 1, Figure S1E). The mouth opens during the pretorsional veliger stage (additional file 1, Figure S1H, S1J) whereas the anus opens in the late posttorsional stage at the site of a few ciliated cells (anal markers). The development of the digestive tract is very similar to that described in *G. cineraria *and *Haliotis tuberculata *[57,58]. The digestive gland begins to differentiate on the left side of the veliger just before torsion sets in [57,58]. The gut develops from differentiated endodermal cells initially scattered within the yolk in the pretorsional veliger. They later migrate to the yolk boundaries to form the definitive midgut in the posttorsional veliger [57,58]. Later, the hindgut develops from actively dividing cells of the digestive gland migrating to their final positions in the intestine [57,58]. The competent larva's digestive system comprises a mouth opening and a bipartite oesophagus (the anterior part immediately behind the buccal cavity is not effected by torsion, the mid oesophagus includes a portion affected by the torsion), a stomach with the digestive gland, the hindgut leading to the anus that opens into the mantle cavity over the back of the head (additional file 1, Figure S2A and S2B). The radula anlage is a ventral differentiation of the foregut where mesenchym cells aggregate. The radula teeth become visible in the competent larva at the distal end of the radula sheath (additional file 1, Figure S2A and S2B).

### *ParaHox *gene sequences

**Figure 1:**
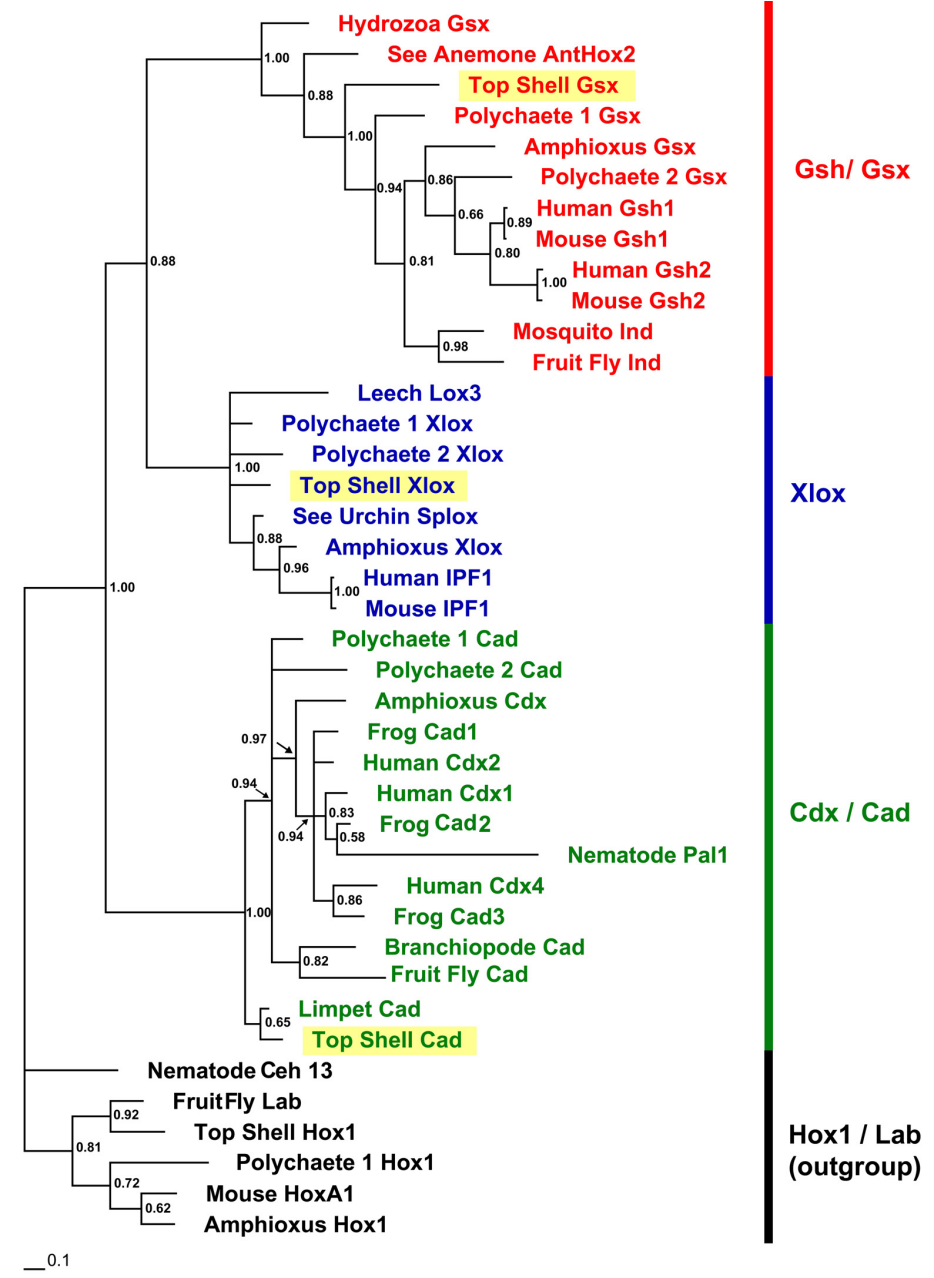
**Phylogenetic reconstruction of *ParaHox *genes**. The tree is from Bayesian likelihood analysis using MrBayes: half compatibility consensus from five million replicates, burn-in of 5,000 replicates. The tree is built with the amino-acid sequences of the homeodomain and the flanking region. Support values of branches are posterior probabilities of Bayesian likelihood. *Hox1 *sequences of several bilaterians are used as outgroup (black). Groupings of the *ParaHox *genes are strongly supported. *Gsx/Gsh *sequences are shown in red, *Xlox *sequences in blue, and *Cad/Cdx *in green. Yellow rectangles highlight *ParaHox *sequences of *G. varia*. Amphioxus: *Branchiostoma floridae*, Branchiopode: *Artemia franciscana*, Frog: *Xenopus tropicalis*, Fruit fly: *Drosophila melanogaster*, Human: *Homo sapiens*, Hydrozoa: *Podocoryne carnea*, Leech: *Hirudo medicinalis*, Limpet: *Patella vulgata*, Mouse: *Mus musculus*, Mosquito: *Anopheles gambiae*, Nematode: *Caenorhabditis elegans*, Polychaete 1: *Platynereis dumerilii*, Polychaete 2: *Capitella teleta*, Sea Anemone: *Nematostella vectensis*, Sea Urchin: *Strongylocentrotus purpuratus*, Top Shell: *Gibbula varia*.

The entire coding sequences for all three *G. varia ParaHox *genes were isolated by a combination of 3' and 5' rapid amplification of cDNA ends (RACE, see Methods). 3' and 5' RACE together yielded a complete cDNA of 885 bp with the complete open reading frame (ORF) of 519 bp (172 amino acids) for *Gva-Gsx*, a complete cDNA of 1739 bp with complete ORF of 1002 bp (333 amino acids) for *Gva-Xlox*, and a complete cDNA of 1466 bp with complete ORF of 976 bp (325 amino acids) for *Gva-Cdx*. Alignments of each *G. varia ParaHox *amino acid sequence to orthologs of other species are shown in additional file 2, Figure S3, S4, and S5. Beside the homeobox which is the main region of conservation between *ParaHox *genes, further conserved domains are the N-terminal domain in *Gsx*, and the hexapeptide motifs just upstream of the homeodomains in both *Xlox *and *Cdx *(Additional file 2, Figure S3, S4, and S5). The classification of the *G. varia ParaHox *genes into their orthology groups is apparent from phylogenetic analyses (Figure 1). The species names and accession number of the genes used in phylogenetic analysis are provided in additional file 2. Although the phylogenetic analysis clearly assigns the *Gibbula paraHox *genes to the *Gsx*, *Xlox *and *Cdx *classes with high support values, the internal grouping remains unclear.

### *ParaHox *gene expression in the trochophore larva

**Figure 2:**
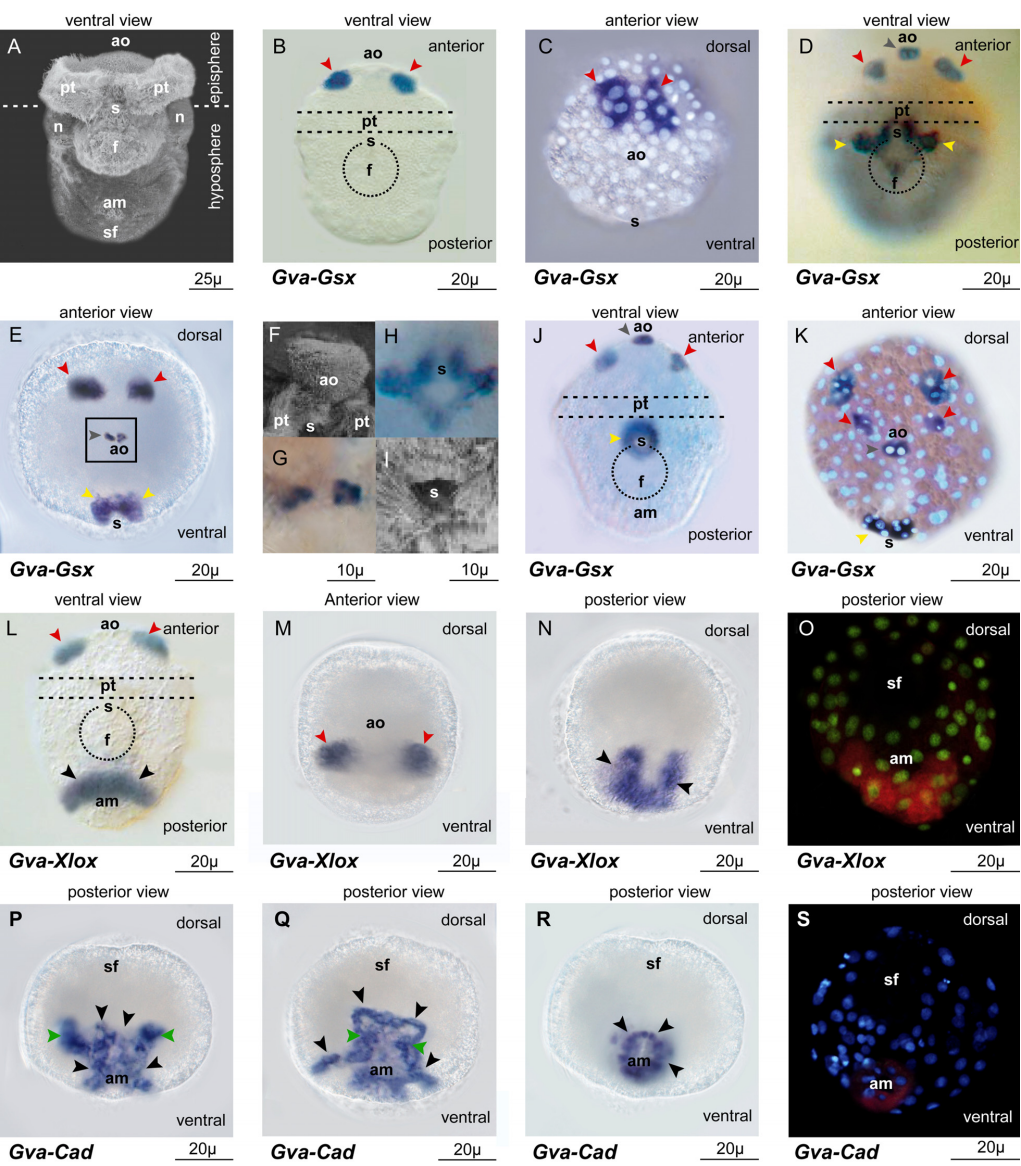
**Expression of *Gva-ParaHox *during trochophore larval stage**. **(A) **SEM of a late trochophore larvae (18-24 hpf). **(B-C) ***Gva-Gsx *expression is first detected in early trochophore larva (12 hpf) in a pair of dorso-medial domains of episphere (red arrow heads). **(D-K) **In late trochophore larva (18-24 hpf) *Gva-Gsx *is expressed in the dorso-medial episphere (red arrow heads), in the apical sensory organ (grey arrow heads), and around the stomodeum (yellow arrow heads). **(F) **is higher magnification of *Gva-Gsx *expression in the apical sensory organ (the area marked by the black rectangle in E) and **(H) **is the higher magnification of *Gva-Gsx *expression around the stomodeum. **(L-O) ***Gva-Xlox *transcripts are detected in a pair of cell clusters in the ventral episphere (red arrow heads) and as a semicircle around the anal marker (black arrow heads). **(P-S) ***Gva-Cdx *is expressed in the left and right primary mesentoblasts (green arrows) and neuroectodermal cells in the hyposphere (black arrow heads). (O) and (S) are false colour images of the in situ hybridization stain, superimposed on fluorescent micrographs stained for nuclei. **am **anal marker, **ao **apical organ, **f **foot rudiment, **n **protonephridium, **pt **prototroch, **s **stomodeum, **sf **shell field.

We did not detect *Gva*-*ParaHox *transcripts by whole-mount in situ hybridization (WMISH) in developmental stages before the trochophore stage. A scanning electron micrograph (SEM) of a late trochophore larva (18-24 hpf) is shown in Figure 2A.

The expression pattern of *Gva-Gsx *is rather dynamic. The first signs of transcripts of *Gva*-*Gsx *are already detected at 12 hpf in early trochophore larvae, when a pair of intensive, bilateral expression domains appears in the dorso-medial episphere (Figure 2B). When viewed from the anterior, each pair of expression domains appears to be composed of 4-5 *Gva-Gsx*-positive cells, presumably in the area of future cerebral ganglia (Figure 2C). This pattern of expression continues in 18 hpf trochophores (Figure 2D and 2E). Here, the pattern of expression becomes considerably more complex. In addition to the paired expression domains in the dorso-medial episphere, *Gva-Gsx *transcripts can now be detected in a pair of cells at the tip of the developing apical sensory organ (Figure 2D and 2E). These two *Gva-Gsx*-positive cells at the tip of the apical organ do not bear any cilia or apical tuft in the trochophore stage of *G. varia *(Figure 2F). The expression of *Gva-Gsx *in the apical sensory organ is restricted to two groups consisting of three sensory cells (Figure 2G). Beside the expression in prospective neural or sensory tissues, *Gva-Gsx *transcripts are also detected around the stomodeum where they appear for the first time in trochophore 18 hpf in two intensely stained bilateral semicircular clusters located anteriorly at the sides of the mouth and a less intensely stained semicircular domain at the posterior part of the mouth (Figure 2D and 2H). Figures 2H and 2I show the trochophore stomodaeum and *Gva-Gsx *expression around it at 18 hpf. About 24 hpf, *Gva*-*Gsx *is expressed in a complete circle around the stomodeum (Figure 2J) and in three episphere domains: a pair of adjacent cells at the tip of the apical sensory organ, and two pairs of cell groups dorsolaterally marking presumptive sites of future cephalic neuroectodermal differentiation (Figure 2K).

*Gva-Xlox *transcription begins later than *Gva-Gsx *expression. No expression is detectable until 24 hpf when *Gva-Xlox *transcripts appear in a group of cells located ventrally in the hyposphere and in a pair of symmetrical expression domains in the medio-ventral episphere of the trochophore larva (Figure 2L and 2M). These symmetrical expression areas are located ventrally of the more intensely stained *Gva*-*Gsx *expression domains in the pretrochal area. *Gva*-*Xlox *is also expressed in the hyposphere in 8-9 cells forming a semicircle around the anal marker (Figures 2N and 2O). These weakly stained *Gva*-*Xlox*-positive cells are probably part of ventral neuroectoderm.

*Gva*-*Cdx *transcripts are first detected in the early trochophore larva (12 hpf). It is expressed at 12 and 18 hpf in two domains in the ventral vegetal plate: one in an area of presumptive posterior neuroectoderm, the other in a bilateral pair of cells in the interior of the larva (Figure 2P and 2Q). Using *Patella vulgata *as a reference, the latter expression of *Pvu-Cdx *probably marks the left and right primary mesentoblasts (green arrows in Figure 2P and 2Q). *Gva*-*Cdx*-positive neuroectodermal cells are first observed as a patch of cells expressing this gene in varying intensities (Figure 2P). Gradually they migrate to the boundary of the expression area (Figure 2Q) so that they from a circle of *Gva*-*Cdx-*expressing cells around the anal marker at 24 hpf (Figure 2R and 2S). The expression of *Gva*-*Cdx *around the anal marker at 24 hpf partly overlaps with the expression of *Gva*-*Xlox *in the ventral area at this stage, which is visible as a semicircle located ventrally around the anal marker (Figure 2N and 2R).

### *ParaHox *gene expression in the pretorsional veliger larva

**Figure 3:**
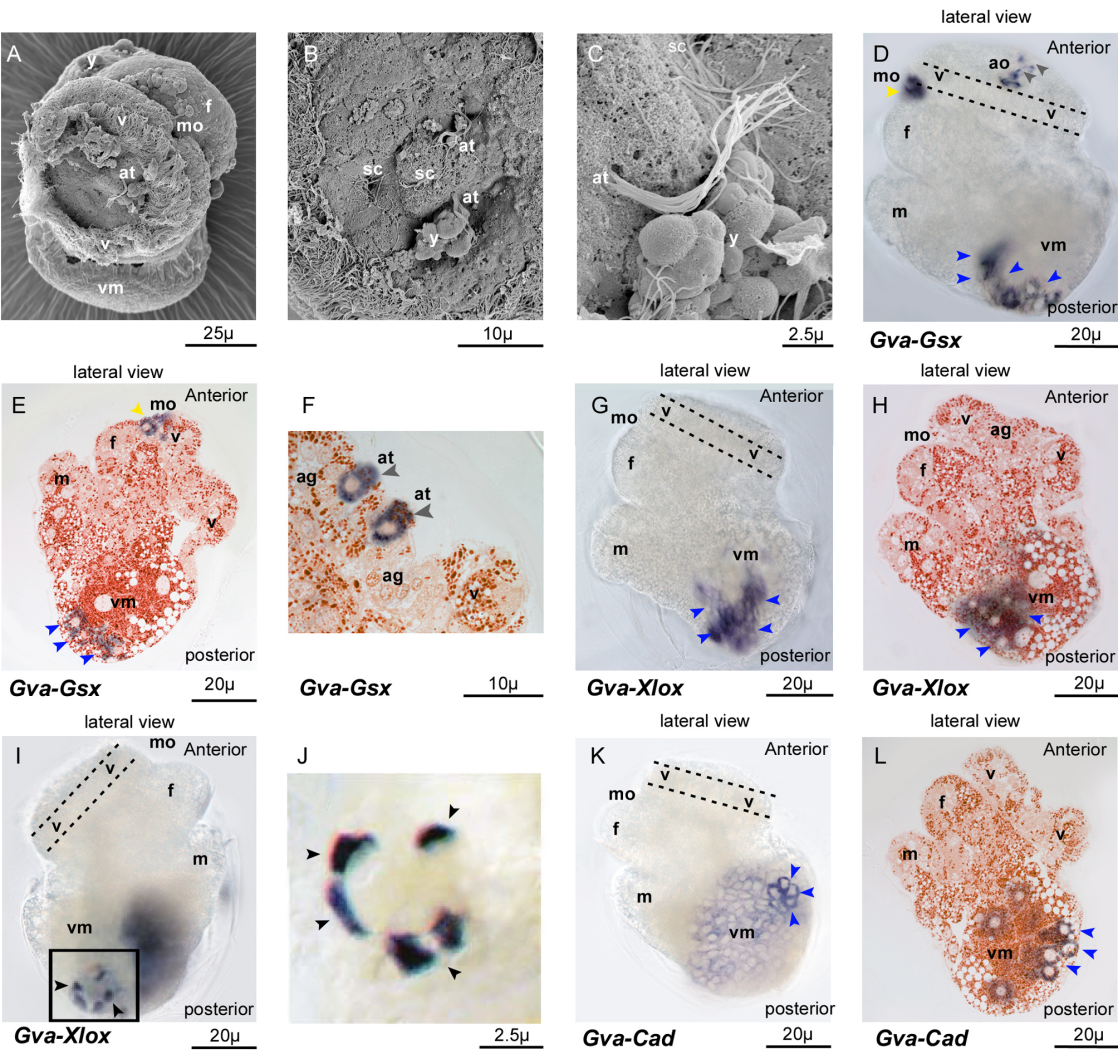
**Expression of *Gva-ParaHox *in the pretorsional larval stage**. **(A) **SEM of pretorsional veliger larva. **(B) **High magnification of SEM of velar area showing the apical tufts and sensory cups. **(C) **High magnification of SEM of ciliated cells of the apical organ. **(D-F) ***Gva-Gsx *transcripts are detected in the area of the apical ganglion (grey arrow heads), in the mouth opening (yellow arrow heads), and in the ventral part of the forming digestive gland in the left side of the visceral mass (blue arrow heads). E and F are histological sections of larvae. (F) is highly magnified expression of *Gva-Gsx *in paired ciliary cells of the apical tuft. **(G-J) ***Gva-Xlox *is expressed in the ventral part of the digestive gland on the left side of larva: (G) and (H), blue arrow heads; and in a group of cells of the ventral neuroectoderm on the right side of the larva: (I) and (J), black arrow heads. The black rectangular area marked in (I) is shown in higher magnification in (J). **(K-L) ***Gva-Cdx *is expressed weakly in the forming digestive gland. The expression intensity is greater in a few cells in the dorsal area of the visceral mass (blue arrow heads). **ag **apical ganglion, **at **apical tuft, **f **foot, **m **mantle edge, **mo **mouth, **sc **sensory cups, **v **velum, **vm **visceral mass, **y **yolk.

The transcripts of all three *Gva*-*ParaHox *genes are detected almost simultaneously in the visceral mass area of the pretorsional veliger larva prior to torsion (36-48 hpf), on the left side of the larva where the digestive gland is forming (Figure 3). At this stage, the velum forms a complete circle and a pair of apical tufts is observed in the velar area (Figures 3A, B, and 3C). In addition to the apical tufts, there are "sensory cups" in the velar area. These are ciliated pockets embedded within the apical ganglion (Figure 3B). The expression of *Gva*-*Gsx *observed in the area of the mouth opening and of the apical organ of the late trochophore larva (Figure 2J and 2K) is retained in the pretorsional veliger (Figure 3D). *Gva*-*Gsx *transcripts are also detected in the ventral part of the forming digestive gland in the left side of the visceral mass (Figure 3D and 3E). *Gva*-*Gsx*-positive signals are further detected in the area of the mouth opening (Figure 3E) and in five cells in the area of the apical organ (Figure 3D), the two apical tuft cells (Figure 3F), and the sensory cup cells (compare Figures 3B and 3D). Similar to *Gva*-*Gsx*, *Gva*-*Xlox *is expressed in the left side of the pretorsional veliger in the forming digestive gland (Figure 3G). The expression area of *Gva*-*Xlox *is located in the ventral part of the digestive gland, more dorsally but partly overlapping *Gva*-*Gsx *expression (Figure 3G and 3H). The expression pattern of *Gva*-*Xlox *detected on the ventral side of the episphere of the late trochophore larva (Figure 2L and 2M red arrow heads) is lost in the pretorsional veliger stage (Figure 3G and 3I). Additionally, five ectodermally derived *Gva*-*Xlox-*positive cells appear on the right side of the larva prior to torsion (Figure 3I). Similar to the trochophore stage (Figure 2M and 2O), these ectodermal cells form an incomplete circle and are presumably linked to the ventral nervous system (Figure 3J). *Gva-Cad *is expressed weakly in the whole area of the nascent digestive gland of the pretorsional veliger larva (Figure 3K). The intensity of expression is stronger in a few cells in the dorsal area of the visceral mass in the left side of the larva (Figure 3K and 3L).

### Expression of *ParaHox *genes in veliger and competent larvae

**Figure 4:**
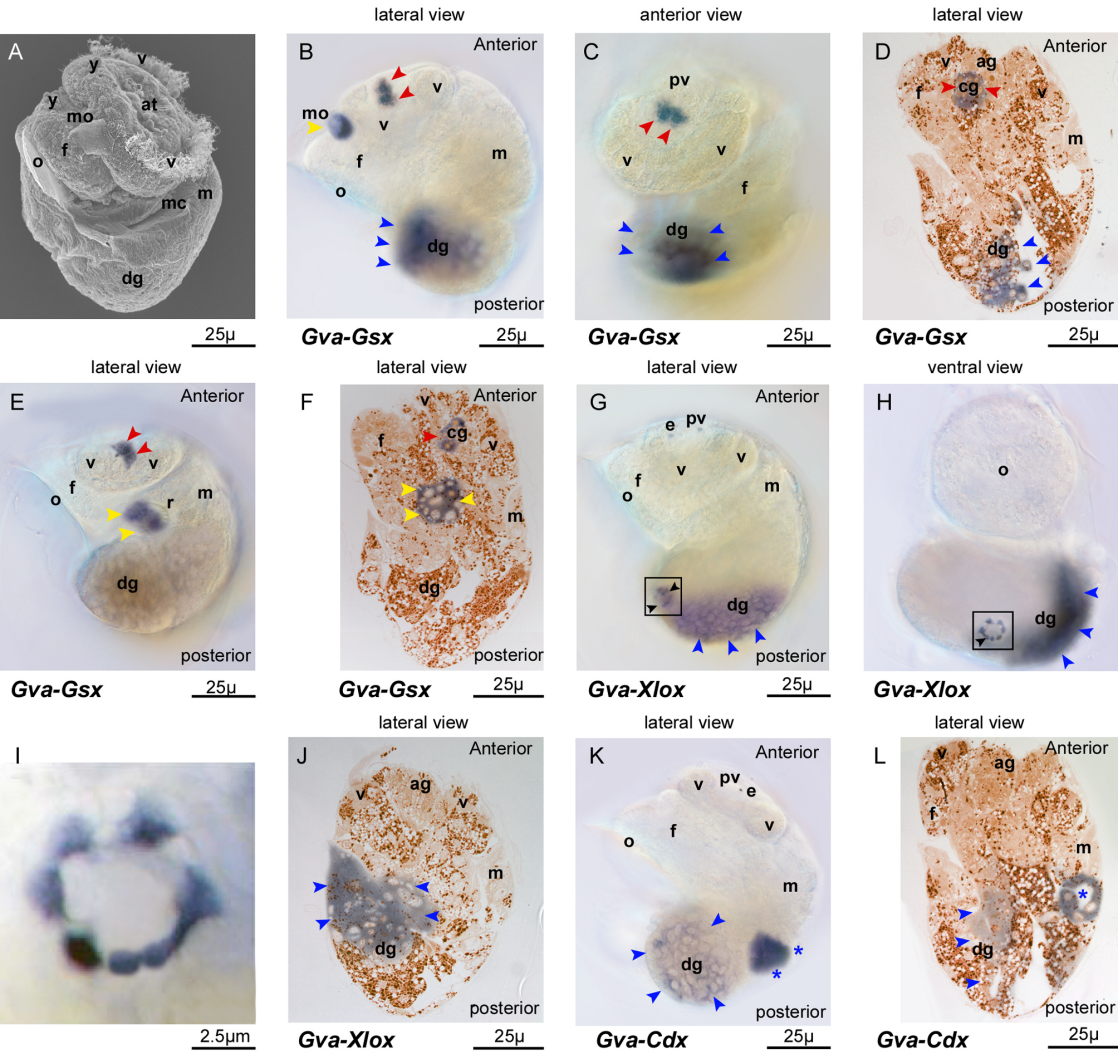
**Expression of *Gva-ParaHox *in the posttorsional larval stage**. **(A) **SEM of pretorsional veliger larva. **(B-D) ***Gva-Gsx *is expressed in the area of the mouth opening (yellow arrow heads), apical ganglion (grey arrow heads), and ventral part of the digestive gland (blue arrow heads). **(E-F) ***Gva-Gsx *expression is detected in the buccal cavity in the forming radula anlage at onset of competence (yellow arrow heads). The gene is also expressed in the forming cerebral ganglia (red arrow heads). **(G-J) ***Gva-Xlox *is expressed in the digestive gland (blue arrow heads) and 6-7 cells of the ventral neuroectoderm (black arrow heads). The area marked by black rectangles in (G) and (H) is shown in higher magnification in (I). A section through the digestive gland is shown in (J). Note that the section is not medial since the digestive gland is located on the left side of the larva. **(K-L) ***Gva-Cdx *is expressed in the hindgut (blue asterisk) and weaker in the digestive gland (blue arrow heads). **ag **apical ganglion, **at **apical tuft, **cg **cerebral ganglion, **e **eye, **f **foot, **m **mantle edge, **mc **mantle cavity, **mo **mouth, **o **operculum, **pv **prevelar area, **sc **sensory cups, **v **velum, **y **yolk.

After torsion (60 hpf), the velum reduces in size with a ventral split, and the mantle expands over the back of the head (Figure 4A). As the digestive tract continues to develop in the posttorsional veliger larva, expression patterns of *Gva*-*ParaHox *become more elaborated. At this stage, *Gva*-*Gsx *expression in the ventral part of the digestive gland and in the area of the mouth opening persists (Figure 4B and 4C). Sections reveal *Gva*-*Gsx*-positive cells at the ventral border of the area of yolk-filled cells (Figure 4D). *Gva*-*Gsx *transcripts are further apparent as paired domains beneath the apical organ where the formation of the cerebral ganglia commences (Figures 4C and 4D). At about three days post fertilization, expression of *Gva*-*Gsx *fades in the digestive gland. Instead, the gene is now expressed in the foregut around the area of the radula anlage (Figure 4E and 4F). At metamorphosis, when the apical sensory organ starts to dissociate, *Gva*-*Gsx *continues to be expressed in the area of the cerebral ganglia (Figure 4F). *Gva*-*Xlox *expression persists on the left side of the visceral mass from the pretorsional to the posttorsional stages (Figure 4G and 4H). Sections through the left side of the larva reveal that these *Gva*-*Xlox-*positive cells are part of the developing digestive gland (Figure 4J). Six or seven ectodermally-derived *Gva*-*Xlox-*positive cells are located in the ventral part of the visceral mass (Figure 4G, H, and 4I). *Gva*-*Cdx *is mainly expressed in the newly formed hindgut and rectum, and weakly in the digestive gland (Figures 4K and 4L).

### Post-larval *ParaHox *gene expression

**Figure 5:**
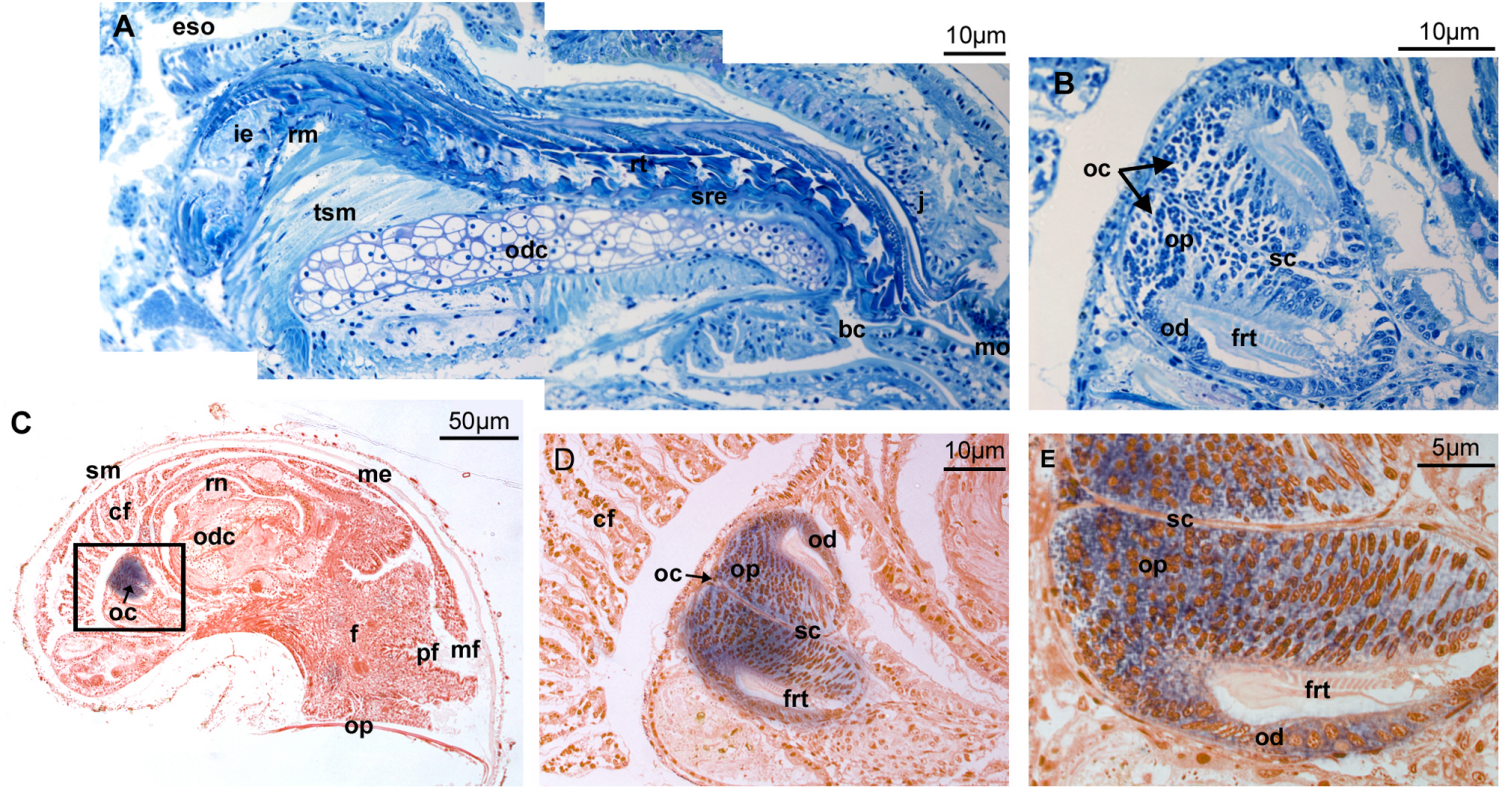
**Post-larval expression of *Gva-Gsx***. **(A) **Medial longitudinal section through the radula of a hatched juvenile stained with Toluidine Blue. **(B) **Paired odontoblastic cushions at the base of the radula sac where radula teeth are formed. The staining is Toluidine Blue. **(C-E) ***Gva-Gsx *expression is observed in odontoblastic cushions of the hatchling. The black rectangular area marked on (C) is demonstrated in higher magnification in (D). The gradient of *Gva-Gsx *expression in odontophores and odontoblast is shown in (E). **bc **buccal cavity, **cf **ctenidial filaments, **eso **esophagus, **frt **forming radula teeth, **ie **inferior epithelium, **j **jaw, **mf **mantle fold, **mo **mouth opening, **oc **odontoblastic cushions, **od **odontoblasts, **odc **odontoblastic cartilage, **op **odontophores, **op **operculum, **pf **pedal folds, **rm **radula membrane, **rn **radula nerve, **rt **radula teeth, **sc **separating cells, **sm **shell matrix, **tsm **tensor muscle.

Serial section in situ hybridizations were used to trace the expression pattern of all three *Gva*-*ParaHox *in the hatchling (about four days after fertilization). No positive signals for *Gva-Xlox *and *Gva-Cad *transcripts are detected at this stage. *Gva-Gsx *is the only *ParaHox *gene that is expressed in the most posterior part of the radula sac during postlarval development (Figure 5). The juvenile hatchling has a complete radula with the radula sheath, buccal musculature, and radula bolsters (also called odontoblastic cartilages, Figure 5A). The posterior end of the radula sac forms the odontoblastic cushion which consists of a single-layered epithelium arranged in a semicircle and protruding into the sac's lumen. The epithelial cells are produced by two separated dorsolateral mitotic centres at the end of the sac (Figure 5B). Mitotic activity is scattered over the posterior area of odontoblastic cushions where the cells are small and undifferentiated. Towards the anterior of the cushions, the cells gradually elongate and form the tall odontoblastic epithelial cells (Figure 5B). *Gva-Gsx *transcripts are mainly detected in the paired odontoblastic cushions at the base of the radula (Figure 5C; the weak signal observed in the pedal area seems to be unspecific). *Gva-Gsx *is expressed both in undifferentiated cells located at the back of the cushions and in odontoblastic epithelial cells. No transcripts were detected in the cells separating the two halves of the odontoblastic cushion (Figure 5D and 5E). The intensity of expression of *Gva-Gsx *diminishes gradually from posterior to anterior, *i.e*. from the undifferentiated cells to fully differentiated epithelial odontoblasts (Figure 5E).

## Discussion

### Is *ParaHox *gene expression colinear during patterning of gut?

It has been proposed that the origin of the three germ layered animals, the Bilateria, is associated with the innovation of several gene clusters of the ANTP family, with the *Hox*-cluster genes participating mainly in patterning of the neuroectoderm, the *NK*-cluster genes in formation of the mesodermal layers, and *ParaHox *in colinear regionalisation of the endoderm [1,3]. Of the animals studied to date, the chromosomal linkage of *ParaHox *genes has been shown only in amphioxus, mouse, and human [1,3]. The *ParaHox *genes are not linked in teleost fishes, the ascidian or the sea urchin [27,28,59]. The only description of the expression patterns of all three *ParaHox *genes for lophotrochozoans in relation to their genomic organisation is for the polychaete *P. dumerilii *[48]. Here, *Gsx *and *Xlox *are clustered and *Cdx *is separated, without clear evidence of colinear expression.

**Figure 6:**
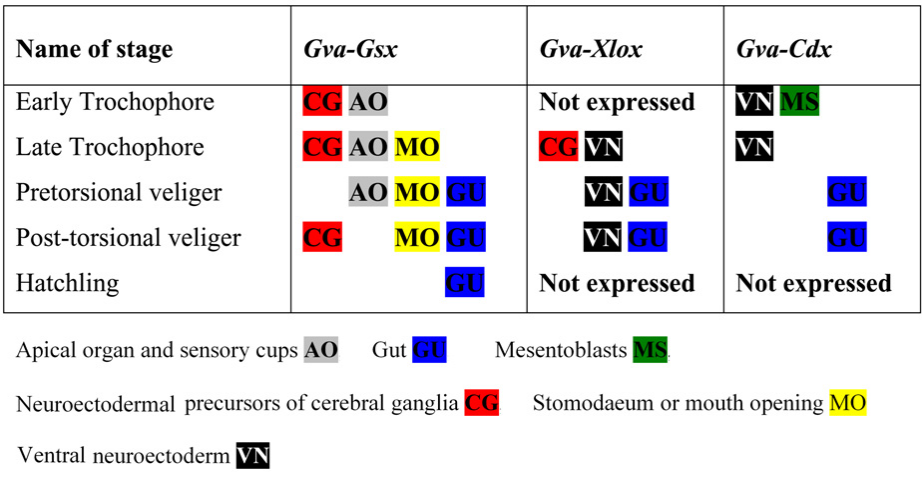
**Summary of Gva-*ParaHox *expression during development of *G. varia***. Note that the colour codes on this figure correspond to the same colours as arrows on the Figure 2-4.

We were unable to detect clear colinear expression of *ParaHox *genes in *Gibbula *prior to torsion. If present, it is obscured by the permanent migration of cells from the digestive gland to their final positions in the gut, and by torsion processes. After torsion, however, a spatially colinear expression of *ParaHox *genes is obvious in the digestive system, with *Gva-Gsx *patterning the mouth opening and radula anlage, *Gva-Xlox *expressed in the midgut, and *Gva-Cdx *in the hindgut (Figure 4 and 6). Therefore, our results support Holland's hypothesis that *ParaHox *genes are involved in gut regionalization along the anterior-posterior body axis in protostomes [3].

There also seems to be a temporal colinearity in expression of *ParaHox *genes in the gradual formation of the digestive system. In the trochophore larva, development of the digestive system begins with the formation of the stomodeum involving *Gva-Gsx *expression only (Figure 6). *Gva-Xlox *and *Gva-Cdx *are expressed at later stages in the more posterior parts of the gut. When the patterning of the gut is completed in the hatchling, expressions of *Gva-Xlox *and *Gva-Cdx *cease while *Gva-Gsx *continues to be involved in the patterning of the radula (Figure 6). During postlarval development, *Gva-Gsx *is expressed in the paired odontoblastic cushions of *Gibbula *(Figure 5). The gradient of *Gva-Gsx *expression from posterior to anterior in the odontoblastic cushions suggests that this gene is associated with mitotic features of these cells and their ability to divide and replace the odontoblasts, rather than direct involvement in secretion of radula teeth.

### Expression of *ParaHox *genes in cephalic neural and neurosensory cells

Gastropod larvae are well provisioned with multicellular sensory structures, but only the apical sensory organ is typically present in both plankton-feeding and non-plankton-feeding veligers [60]. This suggests that information detected by the apical sensory organ is important during the entire larval stage, regardless of the length of larval life or capacity for feeding. Moreover, the apical sensory organ disappears at metamorphosis in species where this has been studied [61]. Therefore the apical sensory organ has functions restricted to the larval stage. During larval development in *Gibbula*, *Gva-Gsx *exhibits a complex pattern of expression in potential cephalic neural cells and in the apical organ. This pattern shows distinct similarity to *Pdu-Gsx *expression in the trochophore stage in which *Pdu-Gsx *expression is detectable in flask-shaped sensory-neurosecretory cells in the medial forebrain [48]. Prior to torsion, the *Gva-Gsx *pattern is spotted in the paired apical tufts and several neurosecretory cells or sensory cups of the apical organ (Figure 3 and 6). This gene also appears to be involved in the formation of parts of the cerebral ganglia from the apical sensory organ in competent larvae. This compares well to the polychaetes *Capitella*, *Nereis*, and *Platynereis*, where *Gsx *is expressed in the cerebral ganglia [45,46,48]. Our results may lend further support to the theory of complex ancestral expression of *Gsx *that was secondarily simplified in several lineages. In addition to *Gva-Gsx *expression in the dorsal episphere of the trochophore, *Gva-Xlox *is detected in a pair of expression domains located more ventrally. It is possible that these cells contribute to neural cells of future cerebral ganglia. However this pattern of expression is transient and is lost in later developmental stages.

### Possible expression of *ParaHox *genes in the trunk neuroectoderm

Expression of *ParaHox *genes in ventral or dorsal neuroectoderm has been demonstrated in several species. Within Lophotrochozoa *Capl-Cdx *is expressed in posterior neuroectodermal cells in the polychaete *Capitella*. In *Platynereis*, *Pdu-Gsx *is expressed in a central part of the larval ventral neuroectoderm in which somatic serotonergic neurons are identified [45,48]. *Nereis *is the only species studied so far in which all three *ParaHox *genes are known to be involved in patterning of the trunk neuroectoderm [46]. In *Gibbula*, *Gva-Xlox *and *Gva-Cdx *are expressed around the anal marker in the trochophore larvae. It has been shown that these cells express *SoxB *in the prospective neuroectoderm of the trunk in *Patella *[62]. Therefore, it is likely that these cells expressing *Gva-Xlox *and *Gva-Cdx *contribute to the trunk neuroectoderm. Temporary expression of *Gva-Cdx *in ventral neuroectoderm earlier during development, and expression of *Gva-Xlox *in overlapping regions at a later stage (Figure 6), may suggest that *Gva-Cdx *contributes to patterning of ventral neuroectoderm upstream of *Gva-Xlox*.

### Hypothetical ancestral *ParaHox *gene expression

Comparative analyses across the animal kingdom show conservation of *ParaHox *gene expression domains in distinct tissues. Comparing *Platynereis ParaHox *gene expression to that of the orthologues in deuterostomes and ecdysozoans, Hui et al. 2009 confirmed Holland's hypothesis about the ancestral role of *ParaHox *genes, suggesting that the pattern of *Gsx *expression in the protostome-deuterostome ancestor was complex, with *Gsx *domains in several structures of the nervous system, and was secondarily reduced to small patches of expression in the anterior CNS in several lineages [48]. Holland's model further suggests that *Gsx *was expressed in the mouth region of the last bilaterian ancestor [3]. Lack of *Gsx *expression in the anterior gut of deuterostomes is explained by loss of the primary mouth and evolution of a new secondary mouth [3]. If this be the case, protostomes should maintain *Gsx *expression in anterior gut structures. *Capitella *results do not support such a model since *CapI-Gsx *expression is limited to a restricted region of the forming brain. The expression of *Nvi-Gsh*, *Pdu-Gsx*, and *Gva-Gsx *described here provides further support to the ancestral mouth patterning role of *Gsx *[46,48].

*Xlox *is expressed during midgut development in annelids [45,46,48-50]. *Pdu-Xlox *and *Nvi-Xlox *are also expressed in the nervous system. In *Gibbula*, *Gva-Xlox *pattern is detected in the digestive gland and ventral neuroectoderm, and expression in potential cephalic nerve cells is transient. Therefore, our results provide additional support that the expression of *Xlox *may reflect an ancestral function in central regions of the gut as well as a role in the nervous system. If this hypothesis is true, however, it would once more imply secondary simplification and loss of neural *Xlox *expression in several lineages [45]. However, the possibility that ancestral *Xlox *expression was simple and has become more complicated in different lineages cannot be ruled out since *Xlox *is expressed in ventral neuroectoderm in *Nereis *and *Gibbula*, in addition to cerebral ganglia, but is lacking in all other protostomes studied to date [46].

*Cdx *shows a complex, dynamic pattern of expression in cells of the ectoderm, endoderm and possibly mesoderm, extending to extremely anterior regions in all annelids studied so far [45-48,51]. This anterior expression of *Cdx *was also recently described in the acoel flatworm, *Convolutriloba longifissura *[63]. *ClCdx *is expressed in the commissures posterior to the statocyst, following the paths of nerve tracks and extending anteriorly. *ClCdx *is also expressed in an area surrounding the eyes, forming direct connections to the brain commissure [63]. *Cdx *anterior expression seems to be the case in the limpet *Patella *as well, in which the gene is expressed in posterior ectoderm during gastrulation. The posterior ectodermal expression starts to fade in the trochophore, while expression extends anteriorly in the shape of an incomplete equatorial ring of ectodermal cells that corresponds to some cells of the prototroch [53]. Later in the young free swimming trochophore, *Pvu-Cdx *expression in the prototroch disappears. The gene is also transiently expressed in the stomodeum [53]. *Gva-Cdx *expression differs from that of *Pvu-Cdx *by being absent during gastrulation. In addition, we did not detect any sign of *Gva-Cdx *expression in the trochophore prototroch or stomodeum. In contrast, the detection of *Cdx *in mesentoblasts and in ectodermal cells situated on the posterior most part of the ventral side of the trochophore is a common feature in *Gibbula *and *Patella*. These are some of the cells that also express *SoxB*, a neurectodermal marker [62]. Therefore, *Cdx *seems to pattern the ventral neuroectoderm as well as mesentoblasts in gastropods. Anterior expression of *Cdx *was not observed during the larval development of *Gibbula *at any stage. This can be either interpreted as secondary loss of the anterior function of *Cdx *in *Gibbula*, or as a gain of function for this gene in several tissues in other species. The first possibility has been favoured since it can be explained by the separation of the gene from the cluster [48]. Nonetheless, variety in the pattern of expression of *Cdx *in different animals can serve as another example for the plasticity of gene expression during evolution. Whether the expression of the *ParaHox *genes in nervous systems is related to their function in the gut, i.e. innervation of different parts of the gut and/or to feeding behaviour, awaits future research. Gene function experiments, therefore, would be desirable to give us better understanding of how these genes are employed.

## Conclusions

The expression of *ParaHox *genes during anterior-posterior development of the digestive system (with *Gsx *patterning the mouth and foregut, *Xlox *patterning the midgut or digestive gland, and *Cdx *patterning the hindgut) suggests that these genes are involved in anterior-posterior specification of the *G. varia *gut. Our results support Holland's hypothesis that *ParaHox *genes are involved in gut regionalization and offer further support to the ancestral mouth patterning role of *Gsx *in protostomes. All three *ParaHox *genes of *G. varia *are involved in patterning of the nervous system. *Gva-Gsx *and *Gva-Xlox *are expressed in neural precursors of cerebral ganglia, the expression domain of these two genes does not coincide in the episphere and fades away in the case of *Gva-Xlox *in later larval stages. Additionally, *Gva-Gsx *patterns the neurosensory cells of the apical organ. *Gva-Xlox *and *Gva-Cdx *pattern the ventral neuroectoderm with *Cdx *possibly acting upstream of *Xlox*. During postlarval development, *Gva-Gsx *transcripts are detected in the precursor cells of odontoblasts at the base of the radula sac. This is probably a molluscan novelty related to radula evolution. Further research in other molluscan classes and use of experimental tools, e.g. RNAi, are required to improve our understanding of gene functions and enable a sound reconstruction of their ancestral role.

## Methods

### Snail culturing

The adults of *Gibbula varia *(L.) were collected in Crete, Greece and cultured in 150-200 liter aquariums in artificial sea water at 22°C (salinity 28°). Copulation was induced by lowering the salinity a few degrees by adding fresh water to the aquariums at 17°C (personal observation of Achim Meyer, The Johannes Gutenberg University of Mainz).

### Cloning of *ParaHox *genes

DNA extraction was performed using the PeqGOLD Tissue DNA kit (PEQLAB Biotechnologie GmbH, Polling, Austria) according to the manufacturer's instructions. Homeobox fragments of *ParaHox *genes were obtained by polymerase chain reaction (PCR) from genomic DNA using *Hox *degenerate primers described previously [64,65]. These primers produce PCR amplification products that are mixtures of different fragments containing homeobox. The PCR fragments were purified using peqGOLD MicroSpin Cycle-Pure Kit (PEQLAB Biotechnologie GmbH, Polling, Austria). Purified PCR products were cloned with the TOPO TA Cloning Kit (Invitrogen GmbH, Karlsruhe, Germany). In total 255 clones were sequenced and all eleven *Hox *genes (Samadi and Steiner, unpublished data) and the three *ParaHox *genes were recovered. RNA was extracted from blastula and gastrula stages, trochophore, veliger, and competent larvae, and encapsulated juveniles using RNeasy Mini Kit (QIAGEN Vertriebs GmbH, Vienna, Austria). The cDNA from each developmental stage was synthesized using SuperScript^® ^III reverse transcriptase (Invitrogen GmbH, Karlsruhe, Germany). The homeobox fragments were used to design primers for rapid amplification of cDNA ends (RACE). The RACE was performed with modifications according to Schramm et al. 2000 [66]. For further details on RACE protocol see supplementary data of [67]. The RACE products were cloned by the Topo-TA cloning kit (Invitrogen GmbH, Karlsruhe, Germany) and sequenced using a BigDye Terminator v3.1 Cycle Sequencing Kit (Applied Biosystems, Foster City, CA, USA) and run on an ABI 3130xl DNA analyser automated capillary sequencer.

### Orthology assignment and phylogenetic analyses

The initial orthology of the *ParaHox *genes was tested by searching against GenBank non-redundant protein databases using the BlastX algorithm. The genes were named *Gva-Gsx*, *Gva-Xlox*, and *Gva-Cdx *and deposited in GenBank under accession numbers HM136802, HM136803, HM136804, respectively. Orthology assignment of the genes was made based on phylogenetic analysis. The phylogenetic analyses were carried out using amino acid sequences. We compiled a *ParaHox *gene alignment including representatives of bilaterians. Sequences were aligned using the program ClustalX v.2.0.10. First the homeobox region was aligned, then, using the homeobox as an anchor, the flanking regions were aligned and subsequent trimming carried out manually. Bayesian inference on amino acid data using MrBayes version 3.1.1 was applied for orthology analysis, with 2 × 4 Markov chains under the Jones amino acid substitution model [68]. Chains were run for five million generations with a sampling frequency of 1000 generations and the burnin set to 5000 generations.

### Whole-mount in situ hybridization

The Maxiscript T7 and SP6 RNA polymerase kit (Ambion, Austin, USA) was used to synthesize the sense and anti-sense probes that were labelled by the Dig RNA labelling kit (Roche Molecular Biochemicals, Vienna, Austria). WMISH was performed with few modifications after Lespinet et al. 2002 [69]. DIG-labelled riboprobes were detected colourimetrically with NBT/BCIP substrates. The details of modifications can be found in [67]. For WMISH, embryos were mounted in 70% glycerol and the expression patterns were documented. For serial-sectioned in situ hybridization, embryos were embedded in Epoxy resin after in situ hybridization according to the standard protocols, and sectioned with a microtome at a thickness of 2 μm. Sections were stained with Eosin using standard histological protocols.

### Scanning-electron microscopy

Larvae were fixed in 4% paraformaldehyde (PFA) in 0.1 M saline phosphate buffer (PBS) for 4 h at room temperature or overnight at 4°C, washed three times for 15 min in PBS containing 0.1% sodium azide (NaN3), postfixed in osmium tetroxide (1% in distilled water for 2 h at room temperature), followed by three washes in distilled water, and dehydrated in a graded ethanol/acetone series. Drying was performed either by critical point dryer or chemical drying with HMDS (Hexamethyldisilazane). After drying, the samples were mounted on scanning electron microscopy (SEM) stubs, sputter-coated with gold, and observed with a LEO 1430VP scanning electron microscope.

## Abbreviations

BCIP: 5-bromo-4-chloro-3-indolyl-phosphate; CNS: central nervous system; hpf: hours post fertilization; HMDS: Hexamethyldisilazane; NBT: nitro blue tetrazolium chloride; ORF: open reading frame; RACE: rapid amplification of cDNA ends; RNAi: RNA interference; SEM: scanning electron micrograph; WMISH: whole-mount in situ hybridization.

## Authors' contributions

LS established the animal cultures, sequenced the *ParaHox *genes, performed WMISH experiments, and wrote the first draft of the manuscript. GS is responsible for the supervision of the project, the phylogenetic analyses, and editing of the manuscript. Both authors have read and approved the final manuscript.

## Supplementary Material

Additional file 1 **General aspect of development and differentiation of gut in *G. varia***. Click here for file

Additional file 2 **Species names and Gene Bank accession numbers of the genes used in phylogenetic analyses and alignments of each *G. varia ParaHox *amino acid sequence to their representatives from other animals**. Click here for file
