# Supplementary material for: Conservation of ParaHox genes' function in patterning of the digestive tract of the marine gastropod Gibbula varia
Source: BMC Dev Biol. 2010 Jul 12;10:74. doi: 10.1186/1471-213X-10-74 (PMC2913954; doi:10.1186/1471-213X-10-74)
Supplement: Additional file 1 — General aspect of development and differentiation of gut in G. varia. [file 1471-213X-10-74-S1.DOC]

### Conservation of *ParaHox* genes’ function in patterning of the digestive tract of the marine gastropod *Gibbula varia*

### Leyli Samadi, Gerhard Steiner

Molecular Phylogenetics, Department of Evolutionary Biology, Faculty of Life Sciences, University of Vienna, Vienna, Austria

### Additional file 1 – General aspect of development and differentiation of gut in *G. varia*


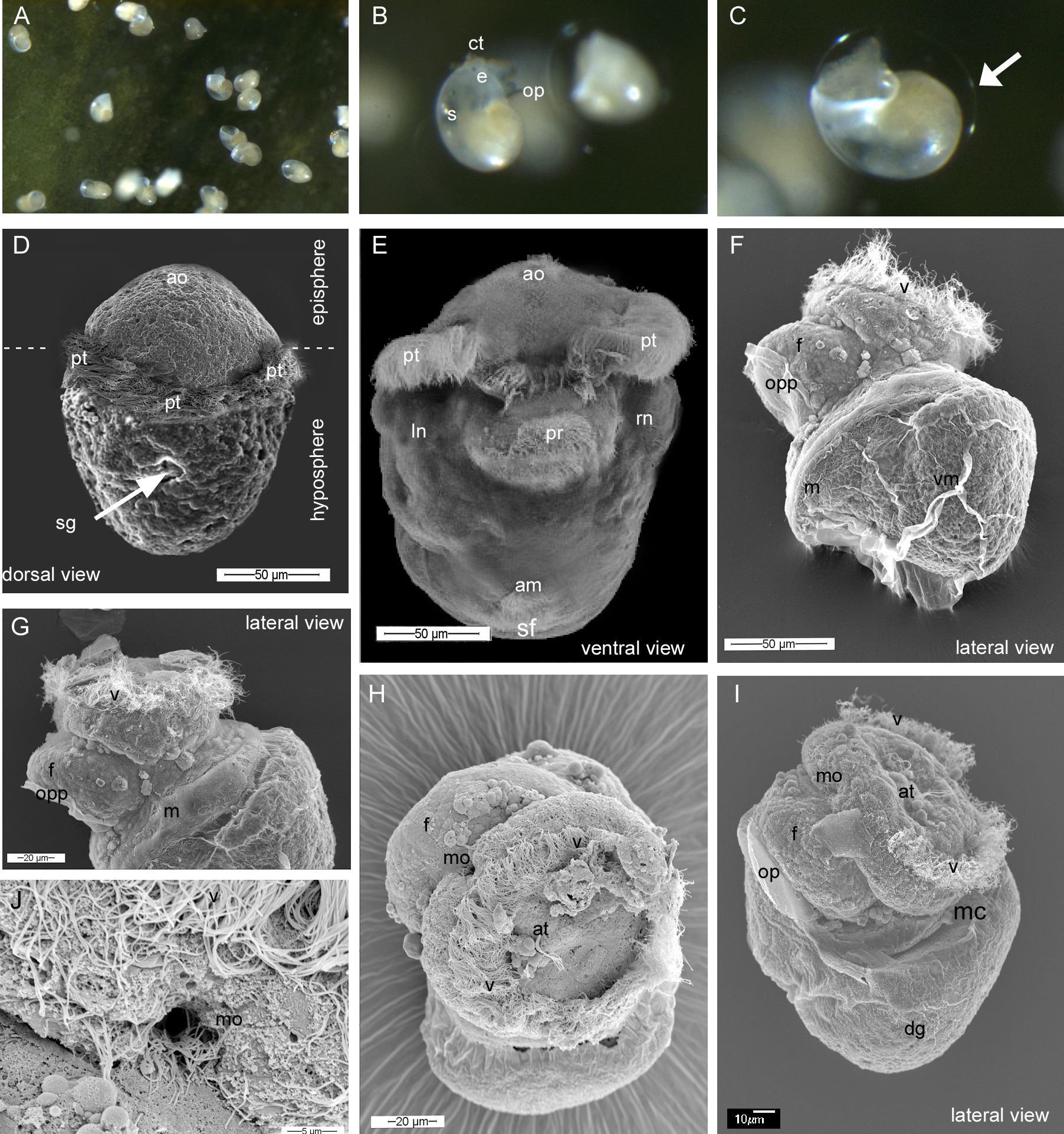


### Figure S1. *Gibbula varia* development. (A-C) A gelatinous egg mass containing encapsulated juveniles. Arrow indicates the capsule surrounding the juvenile. (D) Between 12-16 hpf, the prototroch is clearly visible at the animal pole as a circular ciliary band, separating the trochophore larva to two hemispheres, the episphere (pretrochal) and the hyposphere (posttrochal) regions. The shell gland invaginates at the dorsal posttrochal ectoderm (white arrow). The pretrochal cells are smaller than those of the posttrochal region. (E) Between 18-24 hpf, late trochophore larva comprises a prototroch, the shell field surrounded by the mantle edge, and a pedal rudiment .There is no sign of apical cilia (apical tuft) at any stage in the development of the trochophore. (F-G) Pretorsional veliger larva comprises of a distinct velum, the mantle fold and mantle cavity positioned mid-ventrally, and premordium of operculum. (H) Apical organ is marked by apical cilia (apical tuft) in pretorsional stage. (I) In posttorsional veliger, the velum is split ventrally to form two bands of velar cells that meet around the dorsal region of the head. (J) The mouth opening that is first observed in posttorsional stage is shown in higher magnification. am anal marker, ao apical organ, at apical tuft, ct cerebral tentacle, dg digestive gland, e eye, f foot, ln left nephridium, m mantle, mc mantel cavity, mo mouth, op operculum, opp operculum primordium, pr pedal rudiment, pt prototroch, rn right nephridium, s shell, sf shell field, sg shell gland, v velum, vm visceral mass.


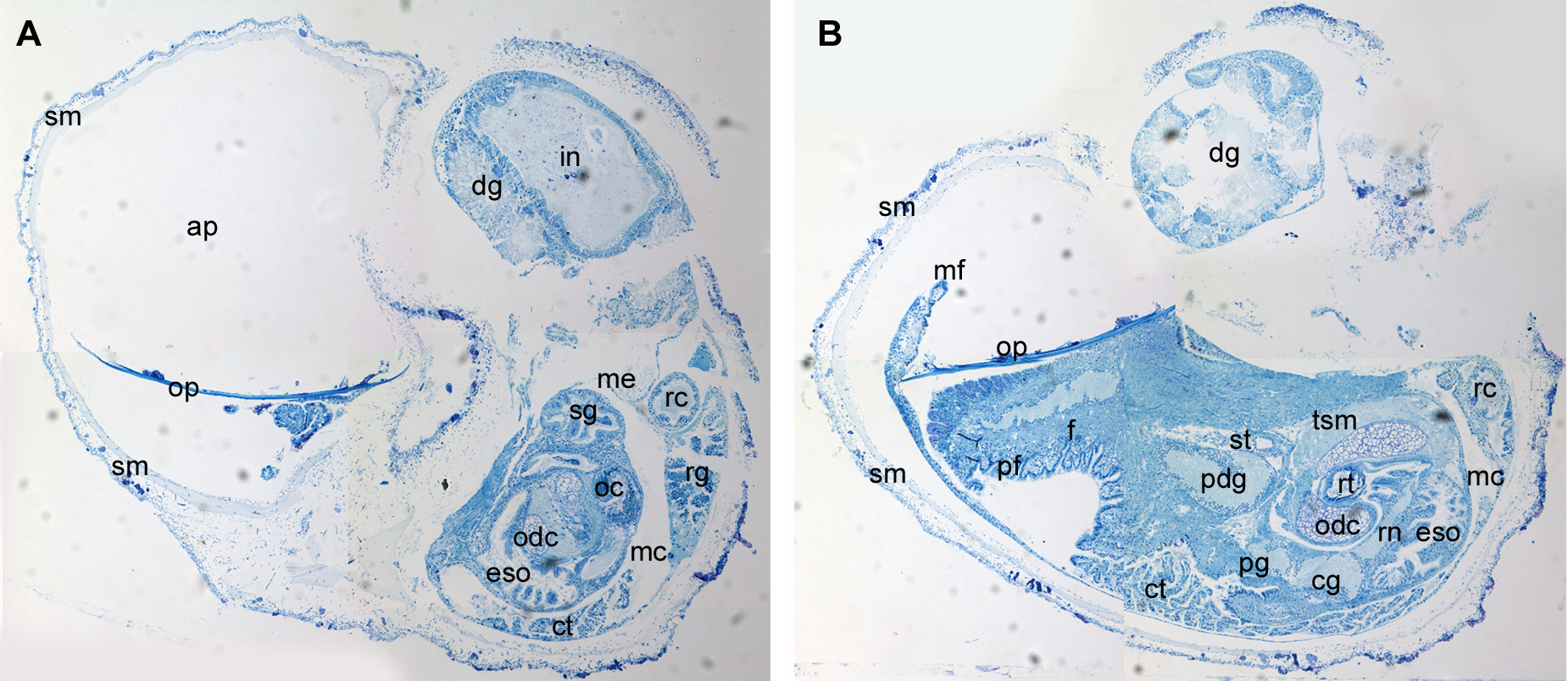


**Figure S2.** Longitudinal semi-thin sections through the encapsulated juvenile of *G. varia* showing the details of digestive system. (A) section through the anterior area. (B) section through the middle of body. **ap** aperture, **cg** cerebral ganglion, **ct** ctenidia, **dg** digestive gland, **eso** esophagus, **in** intestine, **m** mantle, **mc** mantle cavity, **mf** mantle fold, **oc** odontoblastic cushion, **odc** odontoblastic cartilage,

**op** operculum, **pdg** pedal ganglion, **pf** pedal folds, **pg** pleural ganglion, **rc** rectum, **rg** rectal gland,

**rn** radula nerve, **rt** radula teeth, **sg** salivary gland, **sm** shell matrix, **st** statocyst, **tsm** tensor muscle.
