# Supplementary material for: Conservation of ParaHox genes' function in patterning of the digestive tract of the marine gastropod Gibbula varia
Source: BMC Dev Biol. 2010 Jul 12;10:74. doi: 10.1186/1471-213X-10-74 (PMC2913954; doi:10.1186/1471-213X-10-74)
Supplement: Additional file 2 — Species names and Gene Bank accession numbers of the genes used in phylogenetic analyses and alignments of each G. varia ParaHox amino acid sequence to their representatives from other animals. [file 1471-213X-10-74-S2.DOC]

### Conservation of *ParaHox* genes’ function in patterning of the digestive tract of the marine gastropod *Gibbula varia*

### Leyli Samadi, Gerhard Steiner

Molecular Phylogenetics, Department of Evolutionary Biology, Faculty of Life Sciences, University of Vienna, Vienna, Austria

### Additional file 2 – Species names and Gene Bank accession number of the genes used in phylogenetic analysis and alignments of each *G. varia* ParaHox amino acid sequence to their representatives from other animals.

Amphioxus: *Branchiostoma floridae* (*Gsx*: AC129948, *Xlox*: AC129947, *Cdx*: NM_001078201, *Hox1*: BAA78620); Branchiopode: *Artemia franciscana* (*Cad*: CAD98862); Frog: *Xenopus tropicalis* (*Cad1*: AAL14632, *Cad2*: AAL14633, *Cad3*: NP_989417); Fruit fly: *Drosophila melanogaster* (*Ind*: AF095926, *Cad*: AAF53923, *Lab*: CAB57787); Human: *Homo sapiens* (*Gsh1*: AL390738, *Gsh2*: NP_573574, *IPF1*: P52945, *Cdx1*: NP_001795, *Cdx2*: NP_001256, *Cdx4*: NP_005184); Hydrozoa: *Podocoryne carnea* (Gsx: AAG09805); Leech: *Hirudo medicinalis* (*Lox3:* AAB35372); Limpet: *Patella vulgata* (*Cad*: CAD57266); Mouse: *Mus musculus* (*Gsh1*: P31315, *Gsh2*: S79041, *IPF1*: P52946, *HoxA1*: NM_010449); Mosquito: *Anopheles gambiae* (XP_321553); Nematode: *Caenorhabditis elegans* (*Pal1*: 1709361A, *Ceh13*: CAA34929); Polychaete 1: *Platynereis dumerilii* (*Gbx*: EF384214, *Xlox*: FJ001341, *Cad*: DQ188196, *Hox1*: CAD43607); Polychaete 2: *Capitella teleta* (*Gsx*: AAZ23124, *Xlox*: DQ102390, *Cdx*: DQ102389); See Anemone: *Nematostella vectensis* (*AntHox2*: AAD39349), See Urchin: *Strongylocentrotus purpuratus* (*Splox*: AF541970), Top Shell: *Gibbula varia* (*Hox1*: GU056183, *Gsx*: HM136802, *Xlox*: HM136803, *Cdx*: HM136804)


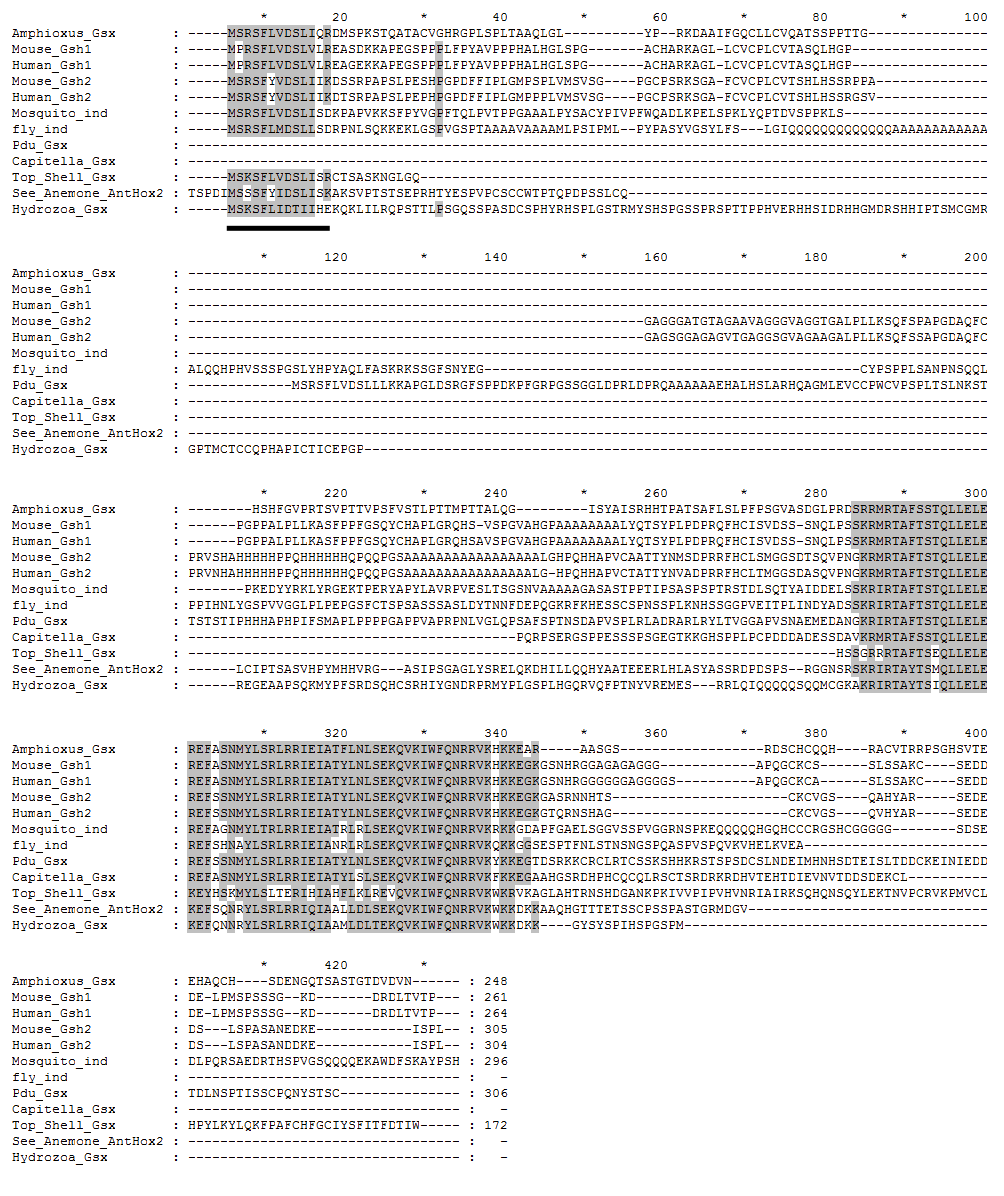


**Figure S3.** Amino acid sequence of *Gva-Gsx* aligned to *Gsx* orthologues from selected taxa. In addition to the largest block of conservation (homeodomain), there is a region of conservation at the N-terminus of the proteins, the SNAG motif (underlined).


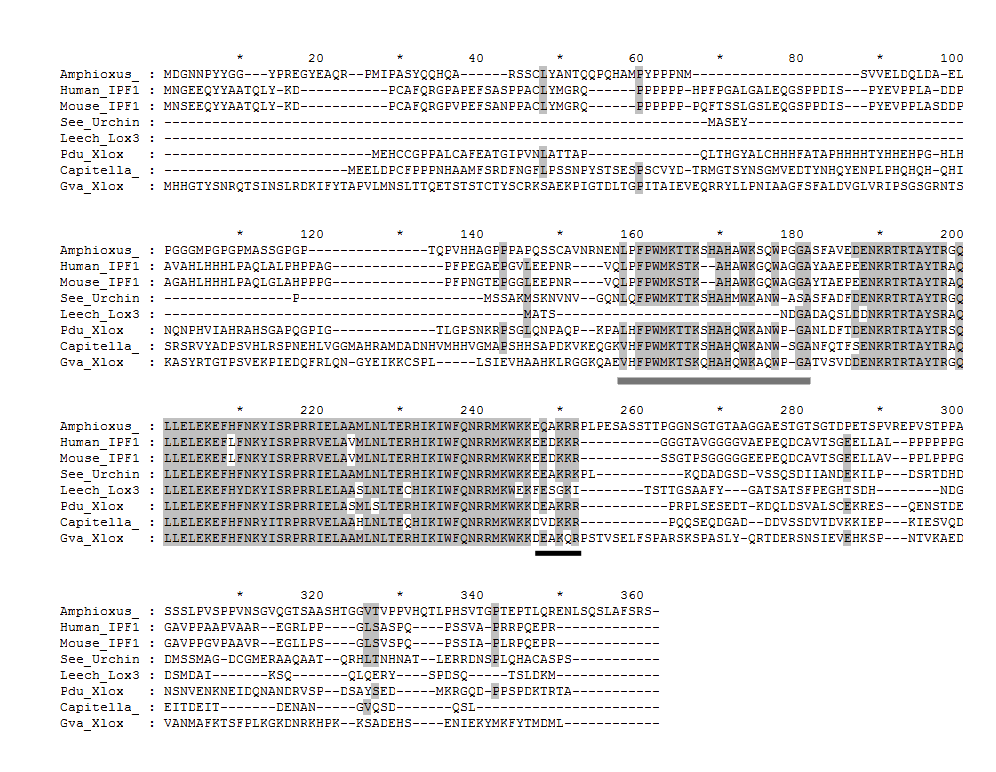


**Figure S4.** Amino acid sequence of *Gva-Xlox* aligned with *Xlox* orthologues from selected taxa. There is a region of conservation just upstream of the homeodomain (marked by a grey bar), and the hexapeptide motif (HFPWMK) (marked by a black bar).


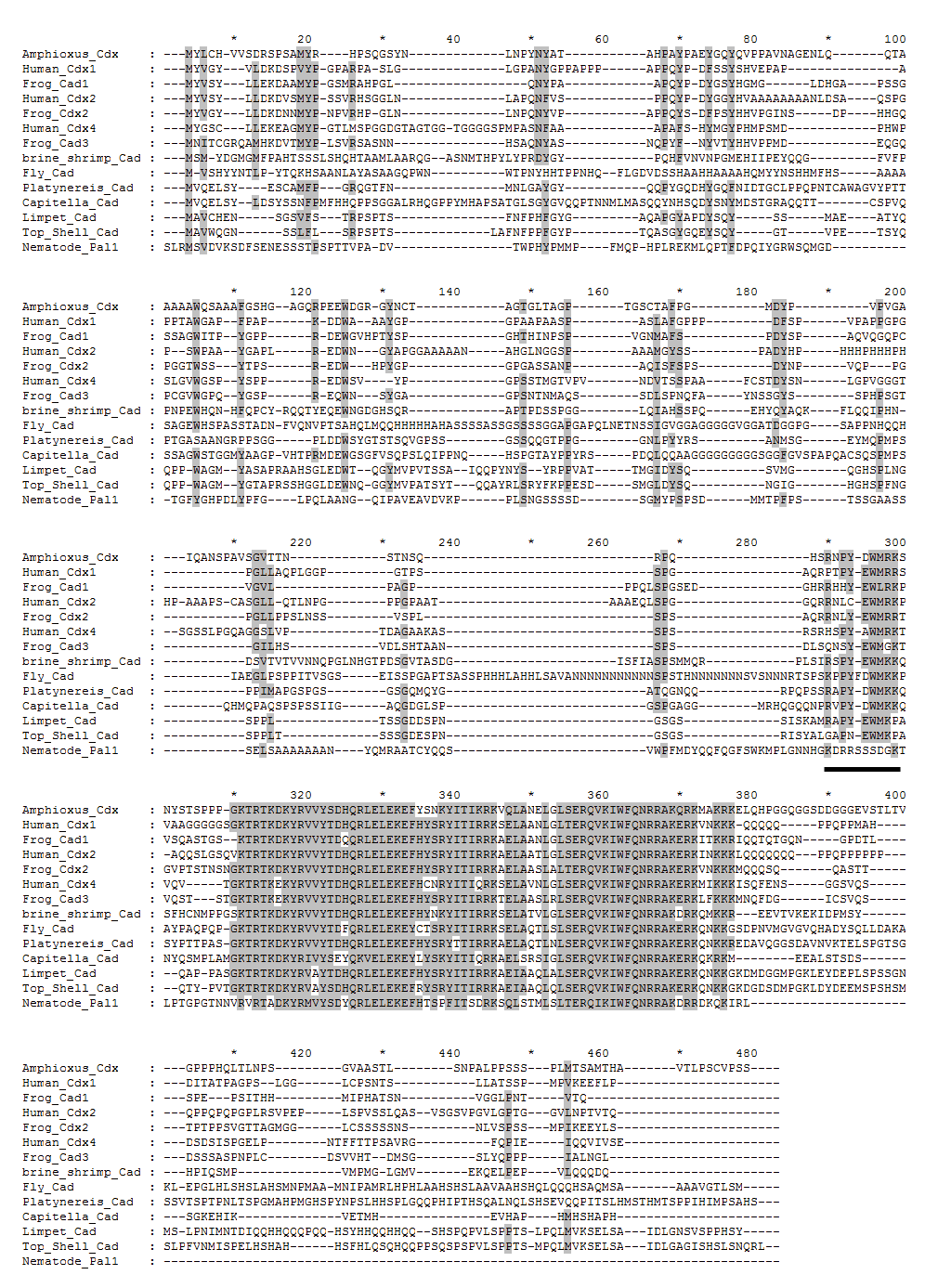


**Figure S5.** Amino acid sequence of *Gva-Cdx* aligned with *Cdx* orthologues from selected taxa. In addition to homeodomain, the largest block of conservation, there is a hexapeptide motif (PYDWMK) just upstream of the homeodomain (underlined).
